# Satisfaction with life in workers: A chained mediation model investigating the roles of resilience, career adaptability, self-efficacy, and years of education

**DOI:** 10.3389/fpsyg.2022.1011093

**Published:** 2022-09-23

**Authors:** Eleonora Topino, Andrea Svicher, Annamaria Di Fabio, Alessio Gori

**Affiliations:** ^1^Department of Human Sciences, Libera Università Maria Santissima Assunta (LUMSA) University of Rome, Roma, Italy; ^2^Department of Education, Languages, Intercultures, Literatures and Psychology (Psychology Section), University of Florence, Firenze, Italy; ^3^Department of Health Sciences, University of Florence, Firenze, Italy

**Keywords:** life satisfaction, well-being, healthy workers, healthy organizations, occupational health, health psychology

## Abstract

Satisfaction with life is a core dimension of well-being that can be of great importance in the workplace, in light of the close link between worker health and organizational success highlighted by the perspective of healthy organizations. This study aimed at analyzing the factors associated with satisfaction with life, focusing on the role of resilience, career adaptability, self-efficacy, and years of education. A sample of 315 workers (67% women; M_age_ = 34.84 years, *SD* = 12.39) filled out the Satisfaction with Life Scale, General Self-Efficacy Scale, Career Adapt-Abilities Scale, the 10-item Connor-Davidson Resilience Scale, and a demographic questionnaire. Data were analyzed by implementing a chained mediation model. Results showed a significant and positive relationship between resilience and satisfaction with life, partially moderated by the chained effect of career adaptability and self-efficacy, controlling for education. When inserted as a covariate, education showed a significant and negative association with satisfaction with life. Such findings contribute to enriching the field of research on the factors that contribute to the well-being of workers and may have important practical implications for interventions in organizations.

## Introduction

The construct of satisfaction with life refers to a conscious cognitive judgment of one’s life and the degree of satisfaction with its current dimensions in comparison to the pursued or ideal quality of life (Diener et al., 1985; Pavot and Diener, 1993). Given its association with both physical and psychological health (Tokay Argan and Mersin, 2021), this construct has found increasing acceptance not only in the clinical literature (Egede et al., 2016) but also in the organizational contexts (Gori et al., 2020). Indeed, the perspective of healthy organizations (Tetrick and Peiró, 2012; Di Fabio, 2017a; Di Fabio et al., 2020; Peiró et al., 2020) underlines the importance of workers’ well-being for organizational profitability, highlighting the strong interdependence between individuals’ health and organizational success. In this view, healthy people are also flourishing and performant workers, able to support and favor the organizational effectiveness and functioning. Therefore, employee’s well-being should be one of the main objectives for organizations, closely related to that of profit (Di Fabio et al., 2020). Consistently, satisfaction with life was associated with a lower risk of occupational injury (Park et al., 2018), lower levels of burnout (Doğan et al., 2015), greater work engagement (Bernales-Turpo et al., 2022), higher levels of job satisfaction (Çevik, 2017), and better job performance (Chughtai, 2021). Given this evidence, analyzing the factors that may impact life satisfaction in workers appears to be of great interest and practical utility. In this regard, the “top-down” perspective (Diener et al., 2003; Steel et al., 2008) emphasizes the role of personal characteristics in contributing to general well-being, highlighting how these can influence both the occurrence of specific events and the ways of interpreting and responding to them (Erdogan et al., 2012). Therefore, following these visions as well as strength-based prevention perspectives in organizations focused on resources (Di Fabio and Saklofske, 2021), the present study aimed to explore the factors that may be associated with satisfaction with life in workers by investigating the effects of resilience, career adaptability, self-efficacy, and years of education.

Resilience is the ability to face the instability of change, persists despite difficulties, and recover positively from failure (Luthans et al., 2006; Senbeto and Hon, 2020). This implies not only being able to cope with adversity, but also the ability to manage them positively and find a new meaning in life (Bonanno, 2004; Gori et al., 2021a, 2021b). Furthermore, resilience has been identified as a core factor for the professional growth of individuals, favoring the acquisition of useful competence for facing challenges at work (Caza and Milton, 2011). Indeed, it has been negatively associated with burnout (McCain et al., 2018), and positively related to both job satisfaction and job performance (Athota et al., 2020); moreover, longitudinal research showed that resilience was a significant predictor of professional skills (Palma-García et al., 2018). Consistently, previous evidence also showed the significant influence of resilience on emotional well-being and satisfaction with life (Liu et al., 2013), which was longitudinally supported (Wang et al., 2022). Furthermore, resilience was also associated with other variables that could mediate this relationship, such as career adaptability (Buyukgoze-Kavas, 2016) and self-efficacy (Djourova et al., 2019).

Career adaptability refers to resources “*for coping with current and anticipated tasks, transitions, traumas in their occupational roles*” (Savickas and Porfeli, 2012, p. 662), successfully managing their professional development (Savickas, 1997) by effectively addressing the pressures and conditions of work environments (Super and Knasel, 1981). Empirical studies have highlighted the association between career adaptability and professional employability (de Guzman and Choi, 2013), self-esteem (van Vianen et al., 2012), job success (Blokker et al., 2019), as well as general and professional well-being (Maggiori et al., 2013). Furthermore, career adaptability was found to be positively predictive of work satisfaction and, in a broader sense, satisfaction with life (Urbanaviciute et al., 2019), but it has also shown longitudinal associations with self-efficacy (Marcionetti and Rossier, 2021), which may intervene in this relationship.

Self-efficacy concerns the individual’s self-assessment of their ability to effectively perform a certain task or their competence in carrying out actions to obtain the desired results (Bandura, 1977, 1993). This aspect determines how the opportunities and environmental barriers are perceived, and influences individual choices and the level of perseverance, thus implying cognitive and motivational aspects related to behaviors linked to both mental and physical subjective well-being (Bandura, 2006a). Consistently, previous evidence showed that self-efficacy was inversely related to the negative impacts of stressors at work (Lee and Ko, 2010) and burnout (Messerotti et al., 2020), while it was positively associated with work performance (Lee and Ko, 2010), as well as job and life satisfaction both cross sectionally (Kondratowicz et al., 2022) and longitudinally (Preetz et al., 2021).

Finally, the level of education appears to be another variable of interest, as it was identified as an important predictor of employability, being married, and health status (Oreopoulos and Salvanes, 2011), elements that could be connected to life satisfaction. In this regard, however, some studies show a positive and statistically significant association between education and life satisfaction (Blanchflower and Oswald, 2004), while others document a significant and negative effect (Powdthavee, 2008). The presence of conflicting results suggests the need to study the phenomenon more in-depth to shed insight into the association between years of education and satisfaction with life in workers.

## Purpose of the present study

In light of this framework, the present study aimed to investigate the relationships between resilience, career adaptability, self-efficacy, and years of education in influencing the levels of satisfaction with life among workers. The latter relationships could be conceived in light of the career construction model of adaptation (Savickas, 2005, 2013; Savickas et al., 2009; Rudolph et al., 2017). The model illustrates that *adaptivity variables* (personality traits, cognitive ability, hope, optimism, or resilience) are associated with career adaptability (adaptability resource; Rudolph et al., 2017). In turn, *career adaptability* and a*dapting response* (e.g., career planning, career exploration, or self-efficacy) partially mediate the relationship between *adaptivity variables* and *adaptation results* (subjective well-being, work performance, and job/life satisfaction). Furthermore, in the model, demographic variables are indicated as *covariates* (Rudolph et al., 2017). Thus, concerning our study’s variables, resilience is an *adaptivity variable*, career adaptability is an *adaptability resource,* self-efficacy is an a*dapting response*, satisfaction with life is an *adaptation result,* and years of education is a *covariate*.

Therefore, a chained mediation model was implemented to test the following hypothesis:

*H_1_*: Resilience would be significantly and positively associated with satisfaction with life.
*H_2_*: Resilience would be significantly and positively associated with both career adaptability and self-efficacy.
*H_3_*: Career adaptability would be significantly and positively associated with self-efficacy.
*H_4_*: Career adaptability and self-efficacy would be significantly and positively associated with satisfaction with life.
*H_5_*: Career adaptability and self-efficacy would consequently mediate the relationship between resilience and satisfaction with life.

Furthermore, since previous studies have shown conflicting results in the effect of years of education on satisfaction with life (Blanchflower and Oswald, 2004; Powdthavee, 2008), the role of education was explored by including this variable in the model as a covariate.

## Materials and methods

### Participants and procedure

The present research involved a sample of 315 workers (211 women and 104 men). Their mean age was 34.84 years (*SD* = 12.39; range 18–61). The inclusion criteria concerned being at least 18 years old and having a good command of the Italian language. All those who did not have a job at the time of completing the survey were excluded (e.g., unemployed, retired, etc.). The recruitment of the participants took place on the Internet, through a snowball sampling. The data was collected online, inviting participants to complete a survey hosted on the Google Forms platform. Each participant provided electronically informed consent. The data were analyzed anonymously and in aggregate form. The Ethical Committee of the Integrated Psychodynamic Psychotherapy Institute (IPPI) approved the protocol of the research before the start of the study (ethical approval number 004/2022).

### Measures

#### Demographics questionnaire

A *Demographic questionnaire* was administered to address information concerning sex, age, marital status (the most recently acquired), education, and the current occupation.

#### Satisfaction with life scale

The *Satisfaction with Life Scale* (SWLS) is a self-report measure designed to assess global life satisfaction (Diener et al., 1985). Participants are asked to answer 5 items on a 7-point Likert scale (from 1 = “Strongly disagree”; to 7 = “Strongly agree”). A sample item is “In most ways my life is close to my ideal.” The total score of the Italian version (Di Fabio and Gori, 2016c, 2020) was used in this research, and its internal consistency measured through Cronbach’s alpha was 0.86.

#### General self-efficacy scale

The *General Self-Efficacy Scale* (GSE) is a self-report measure designed to assess global self-efficacy (Schwarzer and Jerusalem, 1995). Participants are asked to answer 10 items on a 4-point Likert scale (from 1 = “not at all true for me”; to 4 = “very true for me”). A sample item is “I can always manage to solve difficult problems if I try hard enough.” The total score of the Italian version (Sibilia et al., 1995) was used in this research, and Cronbach’s alpha was 0.86.

#### Career adapt-abilities scale

The *Career Adapt-Abilities Scale* (CAAS) is a self-report measure designed to assess the levels of career adaptability (Savickas and Porfeli, 2012). Participants are asked to answer 24 items on a 5-point Likert scale (from 1 = “Not a strength”; to 5 = “Greatest strength”). A sample item is “Thinking about what my future will be like.” The total score of the Italian version (Di Fabio, 2016) was used in this research, and its Cronbach’s alpha was 0.85.

#### Ten-item Connor-Davidson resilience scale

The *10-item Connor-Davidson Resilience Scale* (CD-RISC-10; Campbell-Sills and Stein, 2007) is the 10-item version of the *Connor-Davidson Resilience Scale* (CD-RISC; Connor and Davidson, 2003), a self-report measure designed to assess resilience. Participants are asked to answer 10 items scored on a 5-point Likert scale (from 0 = “Not true at all”; to 4 = “True nearly all the time”). A sample item is “I am able to adapt to changes.” The Italian version (Di Fabio and Palazzeschi, 2012) was used in this research, and its Cronbach’s alpha was 0.94.

### Data analysis

The SPSS software (IBM-SPSS 21.0 version, IBM, Armonk, NY, United States) for Windows was used to analyze the data. The final dataset did not contain missing values, since the used online platform did not allow the submission of surveys unless all items were answered. Descriptive statistics for the sample were calculated. First, the correlation between the variables was explored by performing a Pearson’s r analysis. Then, the chained mediation effect of Career Adaptability and Self-efficacy in the relationship between Resilience and Satisfaction with life, also controlling Education as a covariate, was tested by implementing Model 6 with the macro-program PROCESS (Hayes, 2018). The 95% confidence interval (CI) was calculated for each regression coefficient included in the model and the bootstrapping procedure with a 95% confidence interval (CI) at 5000 samples was used to confirm the statistical stability of the model, supporting the significance of effects when the CI (from lower limit confidence interval [Boot LLCI] to upper limit confidence interval [Boot ULCI]) does not include zero.

## Results

The participants’ sociodemographic information is shown in Table 1. They were predominantly employees (74.0%) and had a high school diploma (42.5%).

**Table 1:** Sociodemographic information of the 315 workers involved in the research.

Characteristics		M ± SD	*N* (*%*)
Age		41.44 ± 12.17	
**Sex**			
	Males		104 (33.0%)
	Females		211 (67.0%)
**Marital status**			
	Single		131 (41.6%)
	Married		115 (36.5%)
	Cohabiting		39 (12.4%)
	Separated		10 (3.2%)
	Divorced		16 (5.1%)
	Widowed		4 (1.3%)
**Education**			
	Middle School diploma (8 years)		14 (4.4%)
	High School diploma (13 years)		134 (42.5%)
	University degree (16 years)		31 (9.8%)
	Master’s degree (18 years)		93 (29.5%)
	Post-lauream specialization (22 years or more)		43 (13.7%)
**Occupation**			
	Employee		233 (74.0%)
	Freelance		41 (13.0%)
	Entrepreneur		21 (6.7%)
	Trader		8 (2.5%)
	Manager		12 (3.8%)

Pearson’s r analysis highlighted significant and positive correlations between all the variables of interest (see Table 2).

**Table 2:** Pearson’s correlation matrix.

	Resilience	Career Adaptability	General Self-Efficacy	Satisfaction with Life
Resilience	1	**0.598** ^**^	**0.681** ^**^	**0.434** ^**^
Career Adaptability		1	**0.523** ^**^	**0.455** ^**^
General Self-Efficacy			1	**0.423** ^**^
Satisfaction with Life				1

Bold indicate significant values.

** Correlation is significant at the 0.01 level (2-tailed).

**Figure 1:**
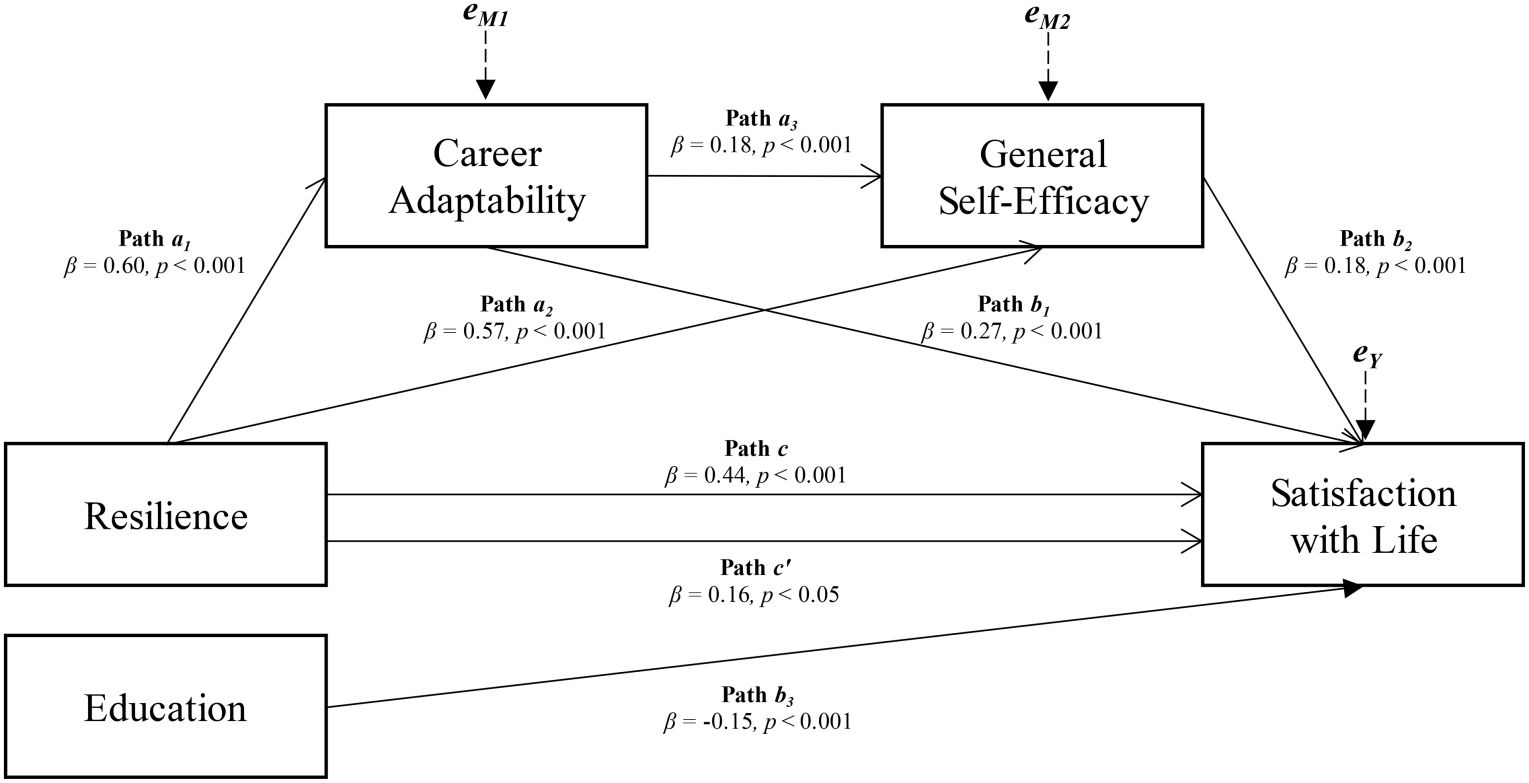
The chained mediation of Career Adaptability and General Self-Efficacy in the relationship between resilience and Satisfaction with Life, controlling for education.

The mediation analysis showed that, controlling for Education, a significant chained mediation model emerged where the positive association between Resilience and Satisfaction with Life was significantly mediated by the consequential effects of Career Adaptability and General Self-Efficacy (see Figure 1).

Specifically, a significant total effect emerged in the association between Resilience and Satisfaction with Life (*β* = 0.44, *p* < 0.001, LLCI = 0.3419–ULCI = 0.5379; H_1_). Furthermore, Resilience was significantly and positively associated with Career Adaptability (*β* = 0.60, *p* < 0.001) and General Self-Efficacy (*β* = 0.57, *p* < 0.001; H_2_). Career Adaptability showed a significant and positive association with General Self-Efficacy (*β* = 0.18, *p* < 0.001; H_3_). In turn, both Career Adaptability and General Self-Efficacy were significantly and positively associated with Satisfaction with life (*β* = 0.27, *p* < 0.001 and *β* = 0.18, *p* < 0.001, respectively; H_4_) and, when were inserted in the model, they partially mediated the association between Resilience and Satisfaction with life (H_5_), which, although reduced, remains significant (*β* = 0.16, *p* < 0.05): *R^2^ =* 0.287, *F*(4, 310) = 31.128, *p* < 0.001. Moreover, Education has been identified as a significant covariate, such as having higher years of education was associated with lower levels of Satisfaction with life (β = −0.15, *p* < 0.001). However, this has not affected the stability of the relationships highlighted in the model, which remained significant also controlling for this co-founder (see Table 3).

**Table 3:** Chained mediation model coefficients.

	Consequents														
		M1					M2					Y			
**Antecedents**		**B**	**SE**	***p***	**95% CI**		**B**	**SE**	***p***	**95% CI**		**B**	**SE**	***p***	**95% CI**
X	*a_1_*	1.523	0.116	<0.001	[1.2948; 1.7516]	*a_2_*	0.437	0.039	<0.001	[0.3604; 0.5133]	*c’*	0.158	0.070	0.025	[0.0196; 0.2955]
M1		–	–	–	–	*a_3_*	0.054	0.015	<0.001	[0.0240; 0.0838]	*b_1_*	0.106	0.024	<0.001	[0.0592; 0.1521]
M2		–	–	–	–		–	–	–	–	*b_2_*	0.234	0.086	<0.001	[0.0644; 0.4039]
C	*a_4_*	0.053	0.570	0.926	[−1.0686; 1.1740]	*a_5_*	0.039	0.153	0.802	[−0.2628; 0.3398]	*b_3_*	−0.718	0.233	<0.001	[−1.1769; −0.2600]
Constant	*i_M1_*	50.628	4.001	<0.001	[42.7562; 58.4989]	*i_M2_*	12.302	1.322	<0.001	[9.6997; 14.9035]	*i_Y_*	4.879	2.275	0.327	[0.4029; 9.3543]
		*R^2^* = 0.358					*R^2^* = 0.484					*R^2^* = 0.287			
		*F*(2, 312) = 86.791, *p <* 0.001					*F*(3, 311) = 97.361, *p <* 0.001					*F*(4, 310) = 31.128, *p <* 0.001			

The model involved Career Adaptability and general Self-Efficacy as chained mediators in the relationship between Resilience and Satisfaction with Life. Education was controlled as covariate. X, Resilience; M1, Career Adaptability; M2, General Self-Efficacy; C, Education; Y, Satisfaction with Life; B, unstandardized coefficient; SE, standard error; 95% CI, 95% confidence intervals.

Finally, the indirect effect was tested through the bootstrapping technique, which confirmed the statistical stability of the model: Boot LLCI = 0.1686—Boot ULCI = 0.4056.

## Discussion

The globalized world of the twenty-first century requires workers to be able to manage the challenges that globalization and technological progress entail (Maree and Di Fabio, 2018; Blustein et al., 2019; Guichard, 2022). Within this context, satisfaction with life has proven to be an important factor linked to job performance (Kołtuniuk et al., 2021), influencing quality, efficiency, and commitment to work (Lado et al., 2021). Therefore, the present research aimed at investigating the variables that may be associated with satisfaction with life in workers, by analyzing the role of resilience, career adaptability, self-efficacy, and years of education.

Results showed a significant chained mediation model, confirming all the hypotheses (H_1_–H_5_). Specifically, a significant total effect was found in the relationship between resilience and satisfaction with life (H_1_). Such findings are consistent with previous evidence (Han et al., 2021) and confirms the importance of resilience as a psychological factor useful for promoting well-being in workers (Richardson and Chew-Graham, 2016). Furthermore, resilience was significantly associated with career adaptability and self-efficacy (H_2_), which in turn were associated with each other (H_3_) and had a significant influence on satisfaction with life (H_4_). This data could be read by the higher tendency shown by resilient people to face and adapt positively to adversity and challenging transitions (Jackson et al., 2007), which can result in effective career management behaviors (Abukhait et al., 2020). Such disposition may be related to greater self-regulation and malleability which allows workers to deal with unknown and complex problems successfully (Savickas and Porfeli, 2012), promoting self-awareness and self-perception as effective (Hartung and Cadaret, 2017; Ginevra et al., 2018; Marcionetti and Rossier, 2021). In turn, higher levels of career adaptability and self-efficacy can be associated with a positive sense of self, even in carrying out new challenges in professional activities (Gori et al., 2022), favoring job success (Hartung and Cadaret, 2017; Carter et al., 2018) and satisfaction with life (Çelik and Kahraman, 2018; Ng et al., 2020). Therefore, the relationship between resilience and satisfaction with life occurred both directly and indirectly through the chained mediation of career adaptability and self-efficacy (H_5_).

Finally, the role of years of education as co-founder was tested, and the model was still statistically solid regardless of this variable, although a significant relationship with satisfaction with life has been found. Specifically, a higher number of years of education was found to be predictive of lower levels of satisfaction with life. This data is part of a frame of conflicting research (e.g., Blanchflower and Oswald, 2004; Powdthavee, 2008). However, more recent evidence shows a significantly negative effect of education on satisfaction with life directly, but also a significantly positive influence when considering the indirect effect involving other factors (e.g., income; Powdthavee et al., 2015). Other evidence that could enrich this framework is the one concerning over-education, that was associated with higher levels of depression, feelings of alienation, and lower job satisfaction (Johnson and Johnson, 2000; Fleming and Kler, 2008; Bracke et al., 2013), and, also in this case, highlighted the importance of considering the income variable, which was found to mediate the negative relationship between over-education and satisfaction with life (Frank and Hou, 2018). Therefore, more studies that include additional variables (e.g., income, over-education, etc.) are needed to investigate in-depth the results of the present study.

Furthermore, this research had some limits that should be addressed. First, this is a cross-sectional design study and this implies that the causal links should be interpreted with caution. Although the relationships tested in the model are supported by previous research, some even longitudinally (e.g., Marcionetti and Rossier, 2021; Preetz et al., 2021; Wang et al., 2022), future research needs to confirm the directionality of these associations through longitudinal designs specifically on samples of workers. Furthermore, the sample of workers collected consists of employees, freelances, entrepreneurs, traders, and managers, and the generalizability of the findings to other occupations requires caution. Therefore, future research should replicate these results in more inclusive samples. In addition, data were collected with self-report measures, which may expose them to reporting biases. The use of a multimodal approach for data collection may help to overcome this issue in future research. Moreover, no data was collected regarding the specific job area of workers. Similarly, the effect of aspects that could influence the subjective experiences of the participants, such as family situations, the possible overlap of marital status, the presence or absence of children, or life events that could have impacted working life, to name a few, was not explored. The exploration of the differences based on detailed job area, as well as the impact of other possible intervener factors on the variables of interest in this study and on their relationships could be an important starting point for future research. Finally, the sample consists of a higher percentage of women, although the male prevalence is still substantial. The use of more balanced samples could be an important challenge for future research to ensure the generalizability of the results for both men and women.

Future research could also examine models that include, in addition to life satisfaction, hedonic aspect connected to work as job satisfaction (Judge et al., 1998), and also eudaimonic aspects of well-being in both working activity, as work meaning (Steger et al., 2012), and in life, as meaning in life (Morgan and Farsides, 2009) and flourishing (Diener et al., 2010). Moreover, future studies could consider that self-efficacy is also domain specific (Bandura, 2006b) and thus it could be interesting to include occupational self-efficacy (Schyns and von Collani, 2002) for specific categories of workers (Di Fabio and Palazzeschi, 2008). Future studies could also include vulnerable workers for whom resources for promoting well-being require particular attention (Di Fabio and Svicher, 2021, 2022; Svicher and Di Fabio, 2021).

## Conclusion

The rapid changes characteristic of today’s globalized world imply the need to be able to effectively manage the uncertainties of a constantly evolving context (Di Fabio and Gori, 2016b; Callanan et al., 2017; Blustein et al., 2019). In this regard, our findings showed a significant chained mediation effect of career adaptability and self-efficacy on the association between resilience and satisfaction with life. It is to be noted that the chained mediation model was controlled for the negative effect of years of education. Thus, in other words, our results highlighted the promising role of career adaptability and self-efficacy in further improving the positive association between resilience and satisfaction with life. The presented results may have interesting practical implications in directing formative and supportive interventions in strength-based prevention perspective (Di Fabio and Saklofske, 2021) focused on early promoting resources (Di Fabio and Kenny, 2016), and may enrich the research framework on the factors that may be central to the workers’ well-being (Di Fabio et al., 2016; Di Fabio and Gori, 2016a; Gori and Topino, 2020; Gori et al., 2021; Topino et al., 2021), according to the perspective of healthy organizations (Di Fabio, 2017a; Di Fabio et al., 2020) and psychology of sustainability and sustainable development in organizations (Di Fabio, 2017b; Di Fabio and Rosen, 2018). Furthermore, the negative relationship between education and satisfaction with life suggests the importance of keeping an eye on the right person-job fit (Edwards, 1991), so that workers over-education (and therefore an imbalance in the Demand—ability fit dimension), as well as an inadequate salary (and therefore an imbalance in the Need—supply fit dimension) can be avoided. These results also call for future reflections on aspects also related to eudaimonic well-being both in life and in relation to work (Dik et al., 2015; Svicher et al., 2022b) including decent work for all workers (Duffy et al., 2016, 2017; Di Fabio and Kenny, 2019; Di Fabio et al., 2021; Svicher et al., 2022a).

## Data availability statement

The raw data supporting the conclusions of this article will be made available by the authors, without undue reservation.

## Ethics statement

The studies involving human participants were reviewed and approved by the Ethical Committee of the Integrated Psychodynamic Psychotherapy Institute. The patients/participants provided their written informed consent to participate in this study.

## Author contributions

AG conceptualized the manuscript. AG and ADF supervised, and tutored ET and reviewed, edited, and wrote the final draft of the manuscript. ET wrote the first draft of the manuscript and ran the statistical analyses. ET, AS, ADF, and AG reviewed, edited, and wrote all the drafts of the manuscript. All authors contributed to the article and approved the submitted version.

## Conflict of interest

The authors declare that the research was conducted in the absence of any commercial or financial relationships that could be construed as a potential conflict of interest.

## Publisher’s note

All claims expressed in this article are solely those of the authors and do not necessarily represent those of their affiliated organizations, or those of the publisher, the editors and the reviewers. Any product that may be evaluated in this article, or claim that may be made by its manufacturer, is not guaranteed or endorsed by the publisher.
